# Gut Microbiota and Bipolar Disorder: Advances in Translational Applications

**DOI:** 10.2174/011570159X379789250626044050

**Published:** 2025-07-17

**Authors:** Liujiao Yan, Shaoxia Zhang, Xiaobing Lu, Zezhi Li

**Affiliations:** 1 Department of Nutritional and Metabolic Psychiatry, The Affiliated Brain Hospital of Guangzhou Medical University, Guangzhou, Guangdong Province, China;; 2 Department of Psychiatry, Dongguan Seventh People's Hospital, Dongguan, China;; 3 Guangdong Engineering Technology Research Center for Translational Medicine of Mental Disorders, Guangzhou, China;; 4 Key Laboratory of Neurogenetics and Channelopathies of Guangdong Province and the Ministry of Education of China, Guangzhou Medical University, Guangzhou, China;; 5 Jiangsu Key Laboratory of Neurodegeneration, Nanjing Medical University, Nanjing, China

**Keywords:** Bipolar disorder, gut-brain axis, gut microbiota, probiotic, emotional regulation, intestinal flora

## Abstract

Bipolar disorder is a severe, recurrent affective disorder that imposes significant pain and burden on both the patients themselves and the social economy. Recent studies have indicated the involvement of intestinal flora in emotional regulation, as well as its close association with the occurrence and progression of diseases such as bipolar disorder. Therefore, conducting comprehensive research on the impact of intestinal microflora and the “gut-brain axis” on bipolar disorder becomes imperative, offering novel insights into its etiology, diagnosis, and treatment options. Consequently, this article provides an overview of the role and potential mechanisms underlying intestinal microbiota in bipolar disorder.

## 1 INTRODUCTION

Bipolar Disorder (BD) is a severe and recurrent mood disorder characterized by both manic and depressive episodes. Globally, the prevalence of bipolar I disorder is approximately 0.6%, while bipolar II disorder is approximately 1.4% [1]. Despite its distinct phenotypic features, BD is often misdiagnosed in clinical settings, resulting in inappropriate and delayed treatment and adverse outcomes [2]. According to the World Health Organization, bipolar disorder ranks sixth among all disease burdens [3]. On average, individuals with bipolar disorder have a life expectancy that is nine years shorter for females and 8.5 years shorter for males compared to the general population, with a twofold increase in all-cause mortality [4]. This is due to the increased risk of chronic diseases such as cardiovascular disease and diabetes, which may result from recurrent symptom episodes, medication treatment, and substance abuse [5].

Although the continuous emergence of various treatment methods for bipolar disorder, the underlying causes remain unclear, resulting in the absence of specific treatment targets. A significant number of BD patients demonstrate inadequate response to monotherapy, necessitating the concurrent use of multiple medications. Consequently, complex medication regimens have become a prevalent practice in clinical settings. The investigation of BD etiology and the development of novel, safe, and effective treatment approaches are currently pressing and pivotal challenges that require immediate attention.

Recent research has revealed that the gut microbiota, through its interaction with the brain *via* the “gut-brain axis”, plays a pivotal role in regulating emotional and cognitive functions, which are closely linked to the occurrence and development of psychiatric disorders, including bipolar disorder. The gut microbiota encompasses intestinal bacteria and other microbial communities, comprising viruses, archaea, and fungi [6]. Individuals with bipolar disorder demonstrate gut microbiota dysbiosis, characterized by reduced microbial diversity and alterations in the abundance of specific microbial taxa. Compared to healthy individuals, those with bipolar disorder exhibit significant disparities in the composition and abundance of their gut microbiota, potentially associated with disease episodes and severity [7]. Furthermore, the gut microbiota may exert an influence on the pharmacological treatment of bipolar disorder [8]. Consequently, a comprehensive investigation into the impact of the gut microbiota and the gut-brain axis on bipolar disorder holds promise for unveiling novel insights into the etiology, diagnosis, and treatment of this condition. This review article aims to provide a comprehensive overview of the role of the gut microbiota in bipolar disorder, including its potential mechanisms.

## 2 GUT MICROBIOTA AFFECTS MOOD, COGNITION AND BEHAVIOR

### 2.1 Gut Microbiota and Gut-Brain Axis

The gut microbiota encompasses both resident microbiota, which colonize the intestinal mucosa, and transient microbiota, derived from ingested food. It is estimated that the gut microbiota comprises over 1,000 distinct species of microorganisms, and each individual's gut microbiota is composed of more than 150 different species [9]. Bacteria dominate the gut microbiota, primarily belonging to the phyla Firmicutes and Bacteroidetes, with additional phyla including Actinobacteria, Proteobacteria, Fusobacteria, Spirochaetes, and Verrucomicrobia. Representative genera within these phyla include Bacteroides, Faecalibacterium, Bifidobacterium, Clostridium, Ruminococcus, and Lactobacillus [10]. Bacterial microorganisms account for 99% of the total microbiota, while fungi, archaea, and viruses comprise the remaining 1% [9, 10].

The major physiological functions of the gut microbiota include maintaining the intestinal epithelial barrier, inhibiting pathogen adhesion to the gut surface, regulating and catalyzing the immune system, and degrading other indigestible carbon sources, such as plant polysaccharides, to produce various metabolites, including vitamins and short-chain fatty acids [11].

Communication between the brain and the gut occurs *via* a network called the gut-brain axis. The gut-brain axis includes the central nervous system, enteric nervous system, autonomic nervous system's sympathetic and parasympathetic branches, neuroendocrine and neuroimmune pathways, and the gut microbiota. Through humoral and neural reflexes, the gut microbiota and the brain construct a complex communication network [12, 13]. Some studies suggest that the intricate relationship between the gut and the brain may affect emotional regulation and have a close association with the occurrence of irritable bowel syndrome and mood disorders, potentially becoming a key target for treating these conditions [14].

**Fig. (1):**
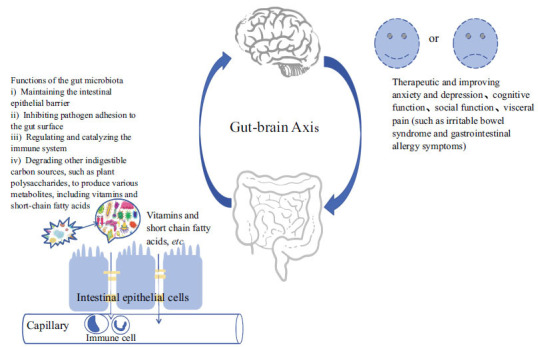
Gut microbiota and gut-brain axis.

Previous research has found that the gut microbiota may have therapeutic and improving effects on anxiety and depression, cognitive function, behavior (primarily social interaction), and visceral pain (such as irritable bowel syndrome and gastrointestinal allergy symptoms) (Fig. **1**).

### 2.2 The Gut Microbiota and Emotional Regulation

Many lines of evidence suggest a link between gut microbiota and emotional regulation. In a chronic mild stress mouse model, it was discovered that the impact of gut microbiota on depressive behavior may be linked to the endocannabinoid system [15]. Needham *et al*. demonstrated that the gut microbiota metabolite 4-ethylphenylsulfate (4EPS) affects brain development and myelination of neurons in mice. Exposure to 4EPS led to anxiety-like behavior in the mice, while drug treatment that promoted oligodendrocyte differentiation rescued the anxiety-like behavior induced by 4EPS. These findings suggest that molecules produced by gut microbiota may affect complex behavior in mice by influencing the function of oligodendrocytes and patterns of myelin phospholipids in the brain [16].

### 2.3 The Gut Microbiota and Cognitive Function

Neurodegenerative diseases characterized by cognitive impairment may be associated with the functioning of the gut-brain axis [17]. Furthermore, research has shown that the gut microbiota plays a crucial role in fundamental processes of neurogenesis, such as blood-brain barrier formation, myelination, neuronal development, and maturation of microglial cells, and it regulates many aspects of animal behavior [18].

In addition, vitamin B may be one of the pathways through which the gut microbiota influences cognitive function. As enzyme cofactors, B vitamins participate in various physiological processes, and clinical data suggest their importance in various psychiatric disorders, including major depression, bipolar disorder, schizophrenia, autism, Alzheimer's disease and Parkinson's disease. Many gut microbiota and bacteria found in fermented foods express genetic mechanisms capable of synthesizing and metabolizing B vitamins, making the gut microbiota rich in probiotics and fermented foods an important source of B vitamins for humans [19]. Increasing evidence suggests that gut bacteria-derived vitamins play a crucial role in physiology, and dysregulation of the “microbiota-vitamin axis” is associated with various diseases.

### 2.4 The Gut Microbiota and Behavior

Recent studies have shown that defects in the gut microbiota significantly impact social behavior in mice. Mice with gut microbiota defects induced by antibiotic treatment exhibit impairments in social functioning. However, the social deficits in mice with gut microbiota deficiency can be improved through interventions such as drugs (including glucocorticoid receptor antagonists and corticosteroid synthesis inhibitors) and adrenal gland surgery. Additionally, genetic ablation of glucocorticoid receptors or chemogenetic inactivation of corticotropin-releasing hormone (CRH)-producing neurons in the paraventricular nucleus of the hypothalamus can reverse social function deficits in mice.

Key microbial taxa that play a role in social behavior have been identified, such as Lactobacillus, which promotes social activity in mice and reduces corticosterone levels under social stress. This suggests that specific gut microbiota may influence social behavior in organisms by modulating stress response pathways and reducing activation levels of the hypothalamic-pituitary-adrenal (HPA) axis [6].

## 3 THE GUT MICROBIOTA IN INDIVIDUALS WITH BIPOLAR DISORDER

### 3.1 Characteristics of the Gut Microbiota in Individuals with Bipolar Disorder

Previous studies have indicated that individuals with bipolar disorder may exhibit characteristic changes in their gut microbiota, which could be implicated in the pathogenesis of bipolar disorder [7]. However, there is still some disagreement within the scientific community regarding the description of specific gut microbiota patterns in bipolar disorder. Meta-analyses have shown that the microbial richness in the gut of individuals with psychiatric disorders, including bipolar disorder, is significantly reduced compared to healthy individuals. In the case of bipolar disorder, this reduction in microbial richness appears to be sustained [7]. Research conducted by Roger S McIntyre *et al*. also confirmed that individuals with bipolar disorder have lower microbial diversity compared to healthy controls [20]. Twin studies have also indicated that affected twins with bipolar disorder have lower gut microbiota diversity and lack specific microbial taxa such as the family Christensenellaceae, and high-risk twins show a trend of decreased diversity. These findings cannot be explained by lifestyle factors such as smoking, alcohol consumption, body mass index, or psychotropic medication usage [21]. Some viewpoints suggest that the depletion of Prevotella and Coprococcus levels and enrichment of Eggerthella levels are consistent in individuals with bipolar disorder, major depressive disorder, and schizophrenia. This suggests that these diseases are characterized by a reduction in bacteria that produce anti-inflammatory butyrate and an abundance of pro-inflammatory bacteria [7]. However, there is still some discrepancy in these research results. A meta-analysis of three studies found lower levels of butyrate-producing bacteria in individuals with bipolar disorder compared to healthy individuals, while another study reported higher levels [22]. Additionally, a study using gene-set enrichment analysis, analyzing whole-genome association data for psychiatric disorders, demonstrated a statistically significant association between the Desulfovibrio genus and bipolar disorder [23]. Previous research has suggested that Desulfovibrio may have toxicity to the intestinal epithelium [24]. Overall, while there is evidence suggesting characteristic gut microbiota alterations in individuals with bipolar disorder, further research is needed to establish consistent patterns and understand the underlying mechanisms. It is a complex and evolving field of study with the potential to provide valuable insights into the pathophysiology and treatment of bipolar disorder.

Other studies have demonstrated greater differences between individuals with bipolar disorder and healthy control groups. It has been found that the phylum Bacteroidetes and the phylum Firmicutes are the main bacterial communities in BD patients and healthy controls, respectively [25]. In a study involving 113 BD patients, 39 unaffected first-degree relatives, and 77 healthy controls, differences in gut microbiota were observed between BD patients and the control group, while no differences were found between first-degree relatives and the control group. The study also revealed differences in the abundance of the genus Prevotella between patients and first-degree relatives [26]. Previous studies have suggested a possible association between high levels of Prevotella and lower quality of life [27]. A meta-analysis reported that four studies identified the families Ruminococcaceae, Rikenellaceae, and Porphyromonadaceae as capable of distinguishing bipolar disorder patients from normal controls [22]. Another meta-analysis found higher levels of Bifidobacterium and Oscillibacter in patients with bipolar disorder [28]. McIntyre *et al*. observed that compared to healthy controls, bipolar participants exhibited a higher abundance of the family Clostridiaceae and individuals with BD-II had higher levels of Collinsella compared to BD-I. Cluster analysis indicated that neither diagnosis nor diet significantly influenced the overall composition of the gut microbiota [20].

**Fig. (2):**
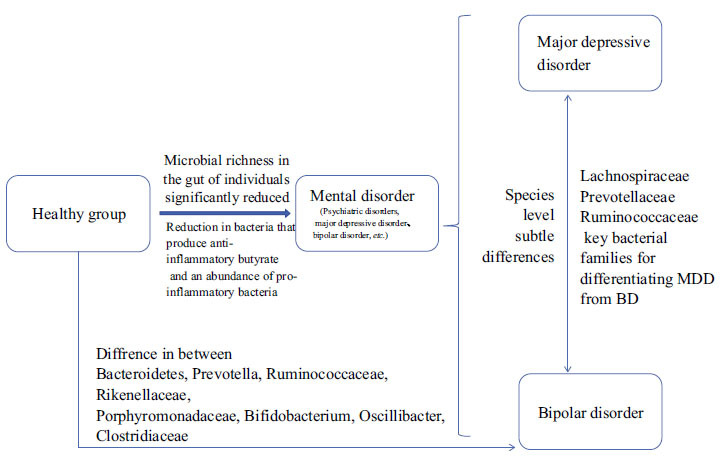
Characteristics of the gut microbiota in individuals with bipolar disorder.

However, in the comparison of three groups: BD, MDD, and healthy controls, it has been found that the relative abundance of the phyla Firmicutes and Actinobacteria increased, while Bacteroidetes decreased in both the MDD and BD groups compared to the healthy control group. At the genus level, when comparing the MDD and BD groups to the healthy control group, it was found that four out of the top five enriched genera (Prevotella, Clostridium, Bifidobacterium, Oscillibacter, and Streptococcus) were significantly increased. The genera Escherichia and Klebsiella showed significant differences in abundance only between the BD and healthy control groups [29]. At the species level, subtle differences in the gut microbiota between BD and major depressive disorder may be crucial for distinguishing between the two diseases. This was confirmed by a study conducted by Zheng *et al*. in 2020, which identified Lachnospiraceae, Prevotellaceae, and Ruminococcaceae as key bacterial families for differentiating MDD from BD [29]. Furthermore, they found that the severity of symptoms was related to the gut microbiota, with four bacteria in the Lachnospiraceae family significantly correlated with Hamilton Depression Rating Scale scores [30]. Some researchers believe that the characteristics of the gut microbiota in BD patients may be gender-specific. They observed a significant increase in three species from the family Lachnospiraceae in female patients, while this pattern was not observed in male patients (Fig. **2**) [31].

### 3.2 The Relationship between Symptoms and Cognitive Function in Bipolar Disorder Patients and the Gut Microbiota

The main symptoms of bipolar disorder include manic episodes and depressive episodes. Regarding manic symptoms, Hamdani *et al*., in a case report of a 46-year-old female patient, mentioned that activated charcoal used to treat intestinal inflammation after gastric bypass surgery significantly improved manic symptoms. This improvement may be attributed to the adsorption of inflammatory mediators by activated charcoal, reducing their impact on the brain after entering the bloodstream [32]. On the other hand, in terms of depressive symptoms, in hospitalized patients with bipolar depression, the association between depressive symptoms and the gut microbiota revealed three distinguishing features: an abundance of Enterobacteriaceae in bipolar depressive patients, and an abundance of Ruminococcaceae and Roseburia in remitted bipolar disorder patients [33]. Zheng *et al*. also found a relationship between the severity of symptoms and the gut microbiota, with four bacterial species in the Lachnospiraceae family significantly correlated with Hamilton Depression Rating Scale scores [30]. Furthermore, in individuals with bipolar disorder, there was a significant correlation between Bifidobacterium counts and improved sleep quality scores, and a significant relationship was observed between lactobacilli counts and sleep [34]. The gut microbiota may modulate immune function to influence cognition and behavior in individuals with schizophrenia and bipolar disorder [35].

### 3.3 The Mechanisms of the Gut Microbiota in Bipolar Disorder

#### 3.3.1 The Interaction between Gut Microbiota and Neuroimmunology in Bipolar Disorder

**Fig. (3):**
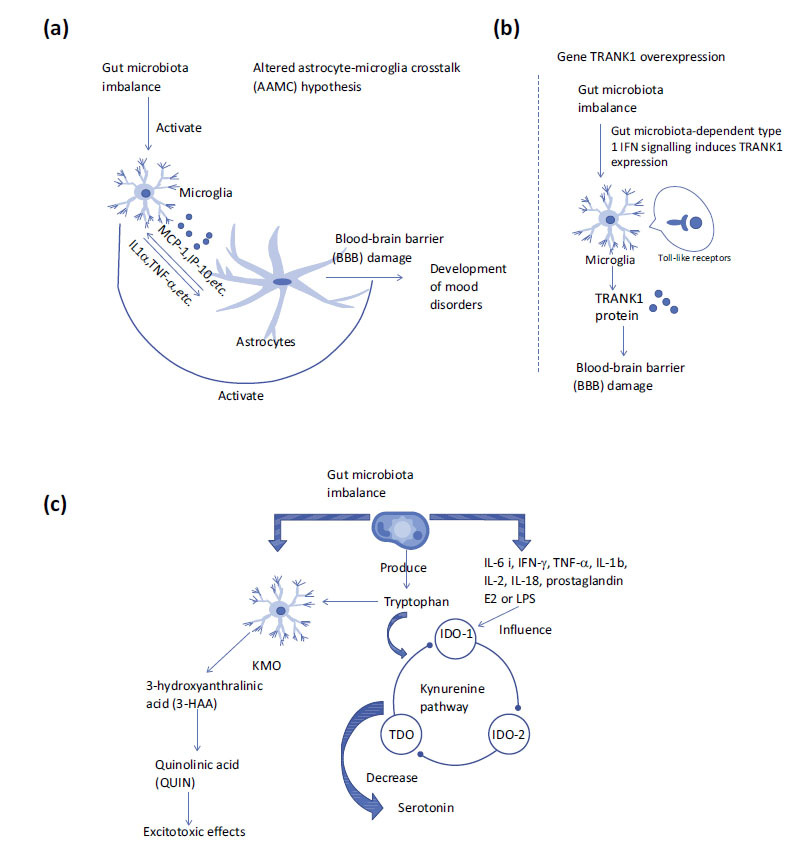
The hypothesis of the gut microbiota in bipolar disorder. (**a**) Altered astrocyte-microglia crosstalk (AAMC) hypothesis. (**b**) The gut microbiota-dependent type 1 interferon (IFN) signaling is sufficient to induce overexpression of the bipolar susceptibility gene TRANK1, leading to compromised blood-brain barrier integrity and facilitating the entry of inflammatory mediators into the brain. (**c**) The increased kynurenine pathway activity may be considered either in the context of tryptophan depletion and reduced serotonin synthesis, or as a cause of increased levels of neuroactive kynurenine metabolites.

The gut microbiota and neuroimmunity in bipolar disorder have been studied, revealing several findings. In comparison to the healthy control group, individuals with bipolar disorder type I exhibit higher IgM reactivity to Morganella morganii, and the IgG to oxLDL is significantly associated with increased bacterial translocation. Through this research, it is proposed that the activated oxidative stress pathway and autoimmune response to oxidative-specific epitopes in mood disorders may be driven by the breakdown of the gut barrier cells, transcellular, and/or vascular pathways [36]. There are multiple hypotheses regarding the neuroimmunity aspect of bipolar disorder, including:

1. The altered astrocyte-microglia crosstalk (AAMC) hypothesis suggests that astrocyte-microglia crosstalk regulates mood changes through immune responses, thereby promoting the development of mood disorders [37].
2. The gut microbiota-dependent type 1 interferon (IFN) signaling is sufficient to induce overexpression of the bipolar susceptibility gene TRANK1, leading to compromised blood-brain barrier integrity and facilitating the entry of inflammatory mediators into the brain. The activated neuroinflammation ultimately contributes to the occurrence and progression of bipolar disorder [38].
3. The role of gut microbiota metabolite “quinolinic acid” and its pathway in immune inflammation and psychiatric disorders: Disruption of gut microbial diversity may lead to increased gut permeability and systemic inflammation, which is also linked to the central nervous system. Pro-inflammatory cytokines induce the formation of neuroactive metabolites through the kynurenine pathway, which is associated with several psychiatric disorders. Existing evidence suggests that the toxicity of quinolinic acid metabolites can be reduced by adding probiotics that can influence pro-inflammatory cytokines [39]. Studies have found significant differences in serum metabolomics profiles between individuals with bipolar disorder and the normal control group. It has also been suggested that metabolic active substances produced by gut microbiota, such as B-group vitamins, quinolinic acid, gamma-aminobutyric acid, and short-chain fatty acids, may influence the occurrence of bipolar disorder symptoms through the gut-brain axis (Fig. **3**) [40].

#### 3.3.2 The Relationship between Gut Microbiota and Epigenetics in Bipolar Disorders

Bengesser *et al*. conducted a study on the relationship between gut microbiota, bipolar disorder, and epigenetics, specifically focusing on the methylation of the clock gene ARNTL. They isolated genomic DNA from fasting blood samples of 32 bipolar participants and sequenced their fecal samples. The results showed a significant correlation between the methylation status of the ARNTL CpG site cg05733463 and bacterial diversity and evenness. ARNTL is known to regulate the transcription of monoamine oxidase A. Diversity of the gut microbiome is the factor that exerts an epigenetic influence on the clock gene ARNTL and is believed to be involved in the pathogenesis of bipolar disorder [41].

### 3.4 Other Gut Microbiota and Bipolar Disorders

#### 3.4.1 Enteric Viruses

While the association between enteric viruses and the brain-gut axis and psychiatric disorders has been rarely studied, enteric viruses have been found to be associated with inflammatory immune-mediated diseases such as ulcerative colitis [42] and diabetes [43] in recent research. Simone *et al*. found that enteric viral-induced inflammation resembles immune responses triggered by gut bacteria [44]. In addition, research on children with autism spectrum disorder (ASD) has identified alterations in the composition of the enteroviral group, specifically noting elevated concentrations of Clostridium, Bacillus, and Enterobacter phages. The enrichment of these bacteriophages is strongly associated with disruptions in viral ecology observed in ASD. Furthermore, modifications in the interactions between gut microbiota and the virome may influence the synthesis pathways of neuroactive metabolites. These findings suggest a potential role of enteroviruses in neuropsychiatric disorders [45].

Furthermore, further exploration of enteric viruses revealed that Coxsackievirus B3, found in enteric viruses, exhibits specific oncolytic activity. Preliminary progress has been made in tumor immunotherapy, as local administration of Coxsackievirus B3 significantly recruits natural killer cells and neutrophils, achieving oncolytic effects through immune responses [46]. The specific mechanisms and pathways involved still require further investigation.

The above studies indicate that enteric viruses play an important role in the intestinal epithelial barrier and systemic inflammation. However, there is limited research on the relationship between enteric viruses and the brain-gut axis, which could be a focus of future studies.

#### 3.4.2 Fungi

Fungi represent a significant biomass in the microbial community, and they are ubiquitous in the human living environment. It has been confirmed that fungi, especially Candida albicans, colonize the human gut shortly after birth [47]. Fungi have an impact on various physiological activities in humans and are even associated with the occurrence of diseases. A case report mentioned changes in the gut mycobiota in patients with anorexia nervosa, where four types of eukaryotic microorganisms never before found in the human gut were detected [48]. Stefano Musumeci reviewed several studies showing an association between specific microbial profiles and health. Fungal communities play a key role in physiological and pathological processes such as immune system training, immune diseases, inflammatory bowel disease, and metabolic syndrome, and may also affect mental disorders [49, 50]. As a well-known opportunistic pathogen, Candida albicans has become the focus of research on the intestinal fungiome. Research by Severance *et al*. suggests that *Candida albicans* may be associated with positive symptoms of schizophrenia. Higher levels of Candida antibodies in males are significantly correlated with more severe positive symptoms of schizophrenia, while probiotics can affect clinical symptoms by lowering antibody levels. This phenomenon is not observed in females [49]. Animal studies have shown that mice colonized with Candida albicans show increased anxiety-like behavior, increased corticosteroid base production and dysregulation, and Endocannabinoid N-arachidonic ethanolamine (AEA)changes. AEA has a negative regulatory effect on the hypothalamic-pituitary-adrenal (HPA) axis and anxiety-like behavior. The study reversed the neuroendocrine phenotype and improved the anxiety-like behavior of *Candida albicans* mice by increasing the level of AEA. These results suggest that *Candida albicans* may affect the occurrence of mental symptoms by affecting the HPA axis and endocannabinoid system.

## 4 THE INFLUENCE OF DIFFERENT CLINICAL INTERVENTIONS FOR BIPOLAR DISORDER ON GUT MICROBIOTA AND CLINICAL SYMPTOMS

### 4.1 Probiotics

A randomized double-blind, placebo-controlled trial involving 423 women in New Zealand showed that the gut probiotic HN001 significantly reduced levels of depression and anxiety compared to the placebo. It also decreased clinically relevant anxiety levels [51]. A 24-week follow-up study demonstrated that the use of Lactobacillus rhamnosus and Bifidobacterium lactis subsp. Probiotics reduced the occurrence and duration of readmission in patients with bipolar disorder [52]. A 3-month follow-up study revealed that probiotics improved cognitive responses to sad mood in individuals with bipolar disorder, suggesting that patients taking probiotics may be less prone to ruminating on sad emotions. Additionally, probiotics were significantly associated with a reduction in manic symptoms as measured by rating scales [53]. However, some studies have reported different findings. In a randomized double-blind, placebo-controlled trial involving 38 inpatients with bipolar disorder type I, conducted over an 8-week period, although there were decreases in Young Mania Rating Scale and Hamilton Depression Rating Scale scores, they did not reach statistical significance, possibly due to the small sample size [54]. Bagga *et al*. divided 45 healthy adults into three groups, receiving either probiotics, a placebo, or no intervention for 4 weeks. They underwent functional MRI scans, and the results indicated that probiotics may be associated with changes in brain activation patterns along with subtle variations in gut microbiota [55]. Most studies confirm the beneficial effects of probiotics in treating bipolar disorder. However, further research is needed to determine the specific types of probiotic strains and their impact on symptoms.

### 4.2 Medications

Previous studies showed that although there were no significant differences between patients not receiving antipsychotic drug treatment at baseline and those receiving antipsychotic drug treatment, women who received antipsychotic drug treatment showed a decrease in gut microbiota diversity compared to women not receiving AAP (atypical antipsychotic) treatment after 14 days [56]. Another study demonstrated that the microbiota composition of patients changed after treatment with quetiapine as a monotherapy [57].

Pu *et al*. conducted a study involving 132 patients with mental disorders (schizophrenia, bipolar disorder, schizoaffective disorder) using olanzapine. After 12 weeks of treatment, compared to the placebo group, the berberine group showed a significant decrease in the abundance of Firmicutes and Enterobacteriaceae, and a significant increase in the abundance of Bacteroidetes. In patients receiving berberine, the abundance of Firmicutes decreased significantly, and the abundance of Bacteroidetes increased significantly. In patients receiving the placebo, there was a significant increase in the abundance of Firmicutes compared to baseline. They found that berberine could modulate the gut microbiota and metabolism of patients with schizophrenia or bipolar disorder and mild metabolic disturbances caused by olanzapine [58].

Sodium valproate can interfere with lipid synthesis and distribution in common gut microorganisms, such as bacteria and fungi in the intestine. Evidence suggests that this interference can occur with a concentration as low as 100 µM of sodium valproate. In patients with bipolar disorder receiving high-dose sodium valproate treatment, the concentration of sodium valproate in the gut may exceed this dosage, and its impact on gut microbiota biosynthesis may contribute to side effects such as obesity [59].

Studies have shown that treatment with lithium carbonate in rats significantly reduced inflammation in the intestinal epithelium. They further confirmed that this improvement was achieved by regulating the abundance of gut microbiota and increasing the presence of bacteria producing short-chain fatty acids [8].

### 4.3 Fecal Microbiota Transplantation

In diseases such as irritable bowel syndrome and Crohn's disease [60], fecal microbiota transplantation (FMT) is an emerging therapeutic approach for alleviating symptoms. Some reviews have mentioned the potential efficacy of FMT in improving depressive-like symptoms in animal experiments, but this viewpoint has yet to be confirmed through clinical trials [61]. However, in bipolar disorder (BD), there are only a few case reports available, and there is a lack of large-sample randomized controlled studies to establish its effectiveness, which is still under investigation.

Hinton *et al*. reported a case of a female patient with BD who experienced significant symptom relief after receiving fecal microbiota transplantation from her healthy husband. After six transplants, her depressive symptoms disappeared, and she lost 33 kilograms in weight. Following a total of nine transplants, she completely stopped medication, and her clinical symptoms completely resolved [62].

In a male patient with comorbid bipolar disorder and attention deficit hyperactivity disorder (ADHD) who underwent fecal microbiota transplantation, it was found that his emotional symptoms significantly improved compared to before the treatment, and he successfully reduced medication dosage. His weight also decreased by 4 kilograms, and his ADHD symptoms showed improvement. During a follow-up of four months, he discontinued all medications without symptom recurrence [63].

It is worth noting that only in the study by Gordon Parker [63] was the use of antibiotics and colonic lavage mentioned to clear the existing gut microbiota before transplantation. Currently, there is limited evidence regarding the impact of pre-transplant gut preparation on the efficacy of FMT.

### 4.4 Nutritional Supplementation

Research has indicated a parallel association between the “Western” dietary pattern, characterized by high fat and high sugar fast food, and an increased risk of mood disorders. This diet may lead to anxiety-depression-like behavior, cognitive decline and other changes in rats by changing the gut microbiota and inducing neuroinflammation pathways [64, 65]. Conversely, a “healthy” diet, characterized by higher intake of fruits, vegetables, legumes, nuts, whole grains, and quality sources of protein such as fish and/or seafood, is associated with a reduced risk of mood disorders. A review also suggests that bipolar disorder may be associated with a “modern Western lifestyle,” which includes stress, unhealthy dietary patterns, and insufficient physical activity. These factors may contribute to the occurrence of bipolar disorder through mild systemic inflammatory responses [66]. Rachelle *et al*. suggest a link between the Mediterranean-style diet and improvement in depressive symptoms [67]. Some studies even bring out Foodomics as part of the host-microbiota-exposome interplay, it shows that the gut-brain axis and gut microbiota are believed to play a key role in the relationship between nutrition and mood disorders. However, there is currently insufficient clinical research to fully support these claims.

### 4.5 Exercise

**Fig. (4):**
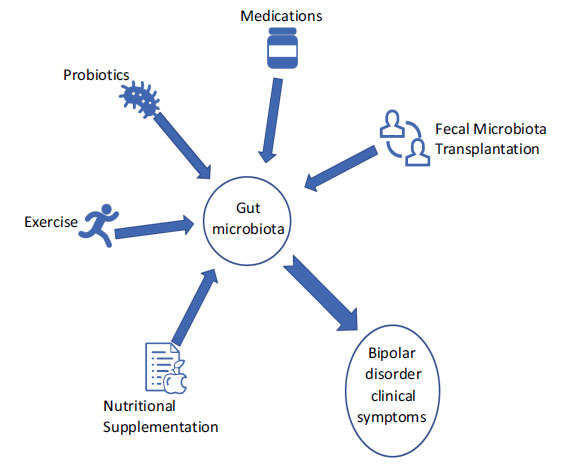
The influence of different clinical interventions for bipolar disorder on gut microbiota and clinical symptoms.

There have been numerous studies confirming the relationship between exercise and stress/anxiety. Exercise is a beneficial adjunctive treatment for bipolar disorder [68, 69]. Recent evidence from mouse models suggests a strong correlation between physical and emotional stress during exercise and changes in the composition of the gut microbiota. Athletes under complex stress conditions are increasingly susceptible to gut-related psychological disorders, which may require adjustments to the athletes' gut microbiota through diet [70]. Aerobic exercise has been shown to increase the diversity and abundance of the phylum Firmicutes, which may be the link between exercise and its positive effects on the gut and brain [71]. Previous research has found that gut bacteria metabolize tryptophan to produce indoleamine 2,3-dioxygenase (IDO), which may play an important role in mental illnesses, leading to the “IDO hypothesis” of tryptophan-indoleamine 2,3-dioxygenase metabolism and its impact on inflammation, metabolism, tumors, and mental disorders. It is proposed that exercise can activate the clearance of indoleamine 2,3-dioxygenase in skeletal muscles to inhibit its accumulation and have an impact on psychiatric disorders such as depression (Fig. **4**) [72].

## 5 DISCUSSION

Bipolar disorder is a challenging mental illness to diagnose and treat, leading to a significant social burden. Delayed diagnosis negatively impacts the progression and treatment of the disease. Therefore, exploring new methods for diagnosing and treating bipolar disorder is a research focus. In recent years, the gut microbiota has emerged as a hot topic, and its influence on mood regulation and cognitive function has gradually been revealed. Previous studies have shown that the gut microbiota may affect brain function through the gut-brain axis. However, the mechanisms by which the gut microbiota acts on the gut-brain axis are not yet fully understood, and its impact on bipolar disorder is still being explored. This review aims to explore the relationship between bipolar disorder and the gut microbiota in terms of symptoms, cognitive function, and neuroimmunity.

The gut microbiota directly affects the central nervous system by producing active metabolites, including B vitamins, indoleamine, gamma-aminobutyric acid, and short-chain fatty acids. Experimental studies have confirmed their impact on the occurrence of various psychiatric disorders, including bipolar disorder, through the gut-brain axis. However, some scholars consider inflammation as the mediator of the gut microbiota's impact on symptoms, and the influence of gut immune response on symptoms may also be an important link in the development of bipolar disorder.

From the above review, it is evident that although the pathological and physiological mechanisms related to the gut microbiota are being discovered, there are still challenges in current clinical trials, such as small sample sizes, unclear controls, and a lack of standardized gut microbiota classification. Moreover, there is a lack of consensus on the gut microbiota characteristics in individuals with bipolar disorder. Additionally, some studies have shown that the decline of butyrate-producing bacterial communities is associated with aging and may be accompanied with the decline in cognitive function [73, 74], which may suggest that age stratification is an important part of the study of gut microbiota in bipolar disorder. Previous studies have primarily focused on adult bipolar disorder patients, and research on adolescent populations is limited. Some studies have demonstrated the interaction between environment and intestinal flora, suggesting regional differences in intestinal flora [75-77]. However, studies comparing the intestinal flora of patients with bipolar disorder in different regions are still lacking. These aspects may provide directions for future research.

Previous studies have shown that interventions such as probiotics and medications can impact both the gut microbiota and clinical symptoms in individuals with bipolar disorder. Exploring comprehensive treatment approaches utilizing multiple intervention strategies may offer new solutions to the treatment challenges faced by bipolar disorder in the future.

## CONCLUSION

Emerging evidence highlights that the gut microbiota plays a key role in the pathophysiology of BD, possibly influencing cognitive function and symptom severity through the gut-brain axis. Microbially derived metabolites such as short-chain fatty acids and B vitamins may mediate these effects through neuroendocrine or inflammatory immune pathways. However, current clinical studies are still limited by small sample size, inconsistent classification of microorganisms, and research subjects are mostly adults, and there is insufficient research on children and adolescents with bipolar disorder. Future studies should prioritize standardized methods, larger cohorts, and the addition of child and adolescent populations to clarify gut microbiota characteristics. In addition, integrating probiotics with interventions such as drugs and exercise may provide new treatment strategies for reducing the burden of bipolar disorder.

## LIST OF ABBREVIATIONS

4EPS: 4-ethylphenylsulfate
AAMC: Altered Astrocyte-microglia Crosstalk
BD: Bipolar Disorder
HPA: Hypothalamic-pituitary-adrenal
